# Comparison of CT and MRI in diagnosis of cerebrospinal leak induced by multiple fractures of skull base

**DOI:** 10.2478/v10019-011-0007-6

**Published:** 2011-03-15

**Authors:** Xuhui Wang, Minhui Xu, Hong Liang, Lunshan Xu

**Affiliations:** Department of Neurosurgery, Research Institute of Surgery & Daping Hospital, Third Military Medical University, Chongqing, China

**Keywords:** CT, MRI, diagnosis, multiple basilar skull fracture, cerebrospinal leak

## Abstract

**Background:**

Multiple basilar skull fracture and cerebrospinal leak are common complications of traumatic brain injury, which required a surgical repair. But due to the complexity of basilar skull fracture after severe trauma, preoperatively an exact radiological location is always difficult. Multi-row spiral CT and MRI are currently widely applied in the clinical diagnosis. The present study was performed to compare the accuracy of cisternography by multi-row spiral CT and MRI in the diagnosis of cerebrospinal leak.

**Methods:**

A total of 23 patients with multiple basilar skull fracture after traumatic brain injury were included. The radiological and surgical data were retrospectively analyzed. 64-row CT (mm/row) scan and three-dimensional reconstruction were performed in 12 patients, while MR plain scan and cisternography were performed in another 11 patients. The location of cerebrospinal leak was diagnosed by 2 experienced physicians majoring neurological radiology. Surgery was performed in all patients. The cerebrospinal leak location was confirmed and repaired during surgery. The result was considered as accurate when cerebrospinal leak was absent after surgery.

**Results:**

According to the surgical exploration, the preoperative diagnosis of the active cerebrospinal leak location was accurate in 9 out of 12 patients with CT scan. The location could not be confirmed by CT because of multiple fractures in 2 patients and the missed diagnosis occurred in 1 patient. The preoperative diagnosis was accurate in 10 out of 11 patients with MRI examination.

**Conclusions:**

MRI cisternography is more advanced than multi-row CT scan in multiple basilar skull fracture. The combination of the two examinations may increase the diagnostic ratio of active cerebrospinal leak.

## Introduction

The incidence of cerebrospinal leak is about 2–9% after the traumatic brain injury.^1^ It is even higher after multiple basilar skull fracture. Secondary intracranial infection, as one of the severe complications, may occur in 30–40% of the patients with prolonged cerebrospinal leak.^2^ A surgical repair is the most effective therapy for most traumatic cerebrospinal leak, which requires the preoperative exact radiological location. The severe craniocerebral injury always results in multiple basilar skull fracture and followed by cerebrospinal leak. The complexity of multiple basilar fractures greatly increases the difficulty of the surgical repair. Preoperatively exact location of cerebrospinal leak is the precondition of surgery, especially in patients undergoing reoperation after the failed surgical repair.

Many examinations have been tried by the radiological experts to diagnose cerebrospinal leak, including radiological cisternography and CT cisternography. However, radioactivity is present in the former, while the latter is time-consuming and the suitable time of scan is hard to choose. Besides, patients may be uncomfortable because of the invasion and the risk of intracranial infection cannot be neglected. With the wide application of multi-row high-resolution spiral CT and MRI, they are being the two main approaches diagnosing cerebrospinal leak. Previous studies have been performed to compare the accuracy of the two examinations in diagnosing cerebrospinal leak. But recently CT and MR techniques are improved greatly. They have not been compared in traumatic cerebrospinal leak, either. Patients with cerebrospinal leak in our hospital were included retrospectively in the present study. The accuracy of CT and MRI cisternography was determined by comparing them with the surgical exploration, so as to provide evidence for the surgical repair of cerebrospinal leak.

## Methods

**FIGURE 1.**
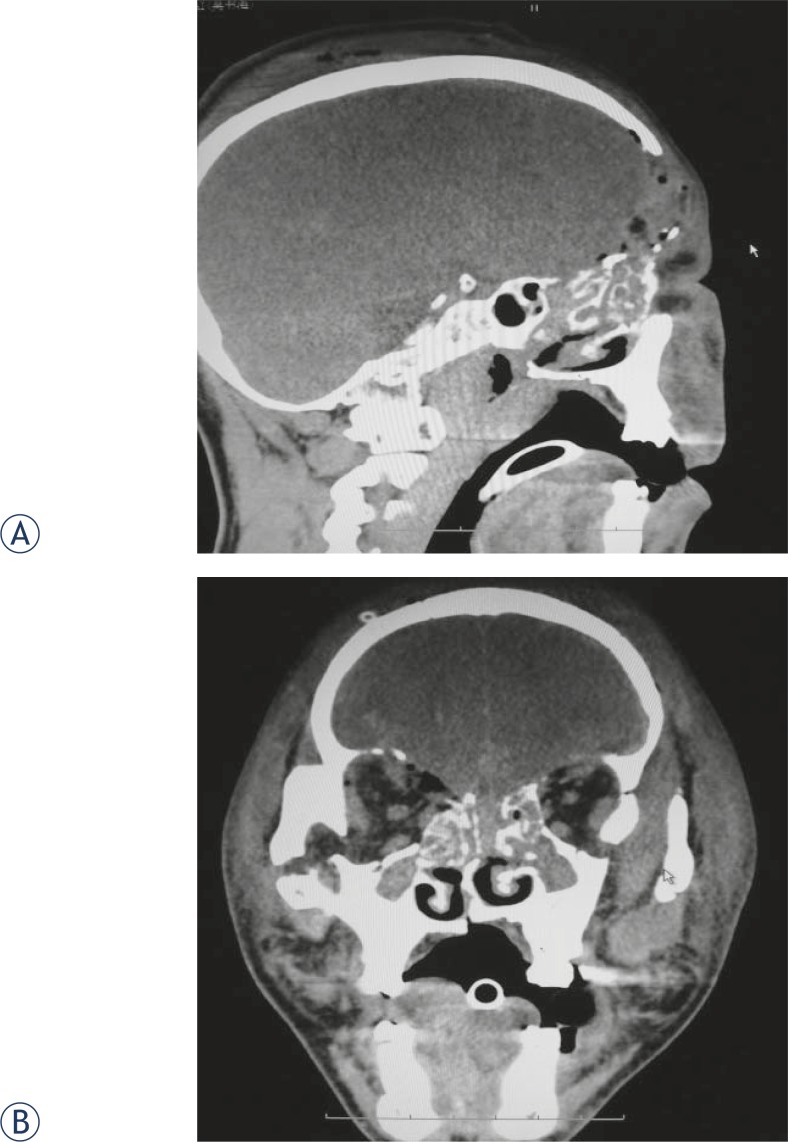
A 43-year-old patient diagnosed with multiple basilar skull fracture induced by severe craniocerebral injury. Partial frontal bone and superficial arch were resected and cerebrospinal rhinorrhea was present 10 days after surgery (Right). CT showed multiple basilar skull fracture. The cerebrospinal leak location could not be determined because of the several defects in ethmoid and sphenoidal sinus. It was demonstrated by surgery that meninges defect was present at the site of ethmoid sinus (A, sagittal view; B, coronal view). Rhinorrhea disappeared after the surgery repair.

Twenty-three patients with traumatic cerebrospinal leak from 2006 in our hospital were retrospectively reviewed. There were 19 males and 4 females, aging from 13 to 40 years, with an average age of 28 ± 3.6 years. All patients were manifested with clear liquid leaking from nose or ear after a trauma, among which rhinorrhea was present in 20 cases and otorrhea in 3 cases. The course of disease was as long as 3 weeks to half a year. Cerebrospinal leak was diagnosed according to the positive ß-2 transferrin in leakage of all patients and finally confirmed by surgery. CT plain scan demonstrated fractures in anterior or middle cranial fossa at 2 or more sites. Then high-resolution spiral CT was performed in 12 patients and MR cisternography was performed in 11 patients. The results of radiological examinations were provided by 2 radiological experts independently. The patient was included in the study only if the 2 radiological experts made a consensus on the diagnosis. The diagnosis was then compared with the actual results identified by the surgical exploration (Figure 1).

High-resolution multiple-row CT scan (64-row, with a thickness of 0.625 mm) was performed in 12 patients. The field of view was 25cm and the matrix size was 512×512. Details of bone substance were shown by the reconstruction. The scanning range included ethmoid, sphenoid and temporal bone, as well as all other sites with potential cerebrospinal fluid leak. 30–50% overlapping was applied for the reconstruction. The site of cerebrospinal leak was suspected when CT scan showed skull base defect, air fluid level and image opacification in adjacent paranasal sinus.^3^

MR cisternography was performed in 1.5T MRI SP 6000 system. T1-weighted imaging in coronal, axial and sagittal planes was scanned as routine. T2-weighted spinecho sequence with fat saturation was obtained in coronal, axial and sagittal planes with parameters of 6000/90/1(TR/TE/excitation). MRI was scanned in both supine and prone position to evaluate the influence of position on the distribution of cerebrospinal fluid. The site of cerebrospinal leak was suspected when cerebrospinal fluid was linked with subarachnoid space outside the skull, or herniation of cerebrospinal fluid was present.

Due to the Chinese system of medical care, the examination fees should be paid by the patients’ own expense. Thus, MR examination was not performed if the diagnostic objective was obtained with CT scan. CT or MR cisternography were recommended by the physicians according to the result of primary CT scan and the medical history. Therefore, there were few direct comparisons of CT and MRI results in the same patient.

## Results

In 12 patients who underwent high-resolution CT examinations, 47 suspected skull defects or fractures were observed and 25 sites of cerebrospinal leak were diagnosed after the analysis: there was a fluid level in the accessory nasal sinuses and the fluid contained glucose and were ß-2 transferrin positive. In comparison with the results of the surgical exploration, 21 sites of cerebrospinal leak were present in 12 patients. A further comparison showed that among 25 sites of cerebrospinal leak, which was seen with the CT examinations, 19 were correctly, 4 was wrong and 2 was missed diagnosed, respectively. The accuracy of CT examination was 90.48%. The diameters of leak site missed diagnosed were about 2 mm, and all missed diagnoses happened in fracture-type defects. Fracture was present in all wrongly-diagnosed sites but no leak was found and diagnosis of cerebrospinal leak was denied during surgery (Table 1).

**FIGURE 2.**
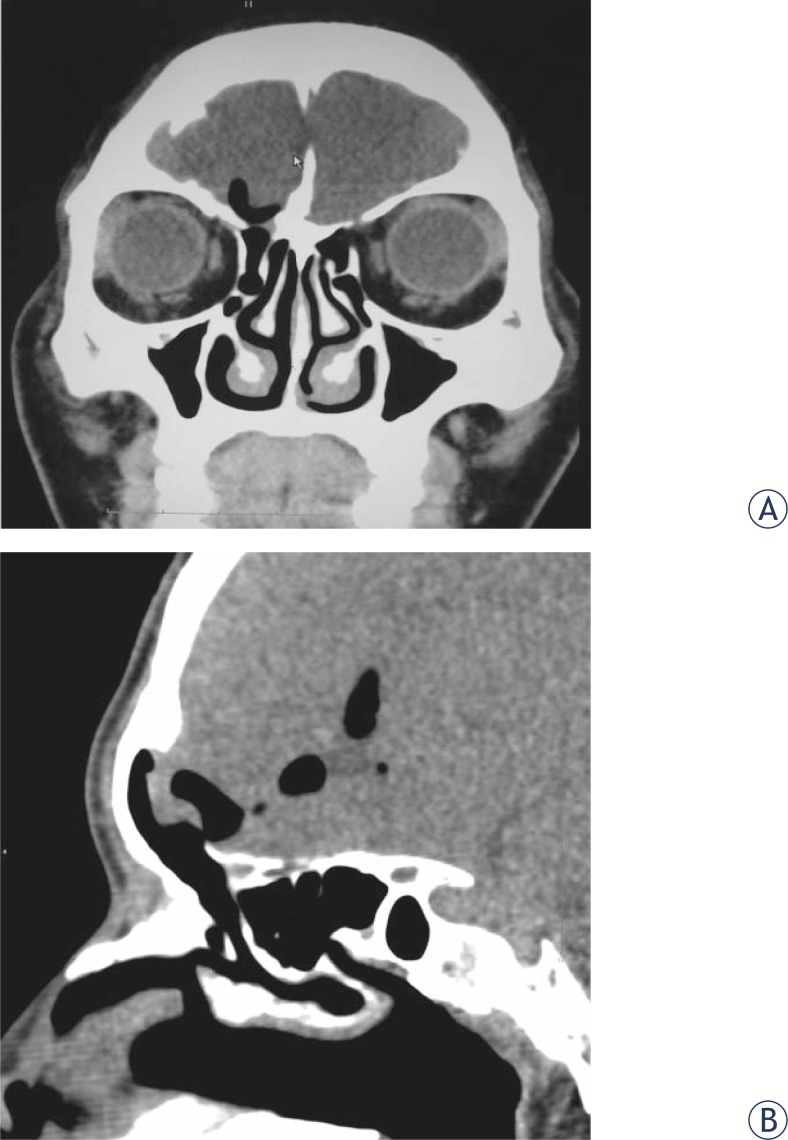
Cerebrospinal fluid rhinorrhea was present after brain trauma in a male patient of 37 year old. CT scan showed multiple basilar skull fracture. Thin layer scan of high-resolution CT showed that frontal sinus communicated with nasal cavity and cerebrospinal leak in frontal sinus was the diagnosis (A, coronal view; B, sagittal view). The surgical exploration confirmed that frontal sinus was impaired and communicated with intracalvarium and rhinorrhea disappeared after the surgical repair.

MR cisternography was performed in 2 patients who underwent CT examination because of the disagreement of the diagnosis between the 2 neuroradiologists. Surgery failure was present in 1 patient after CT examination, in whom cerebrospinal leak in frontal sinus was diagnosed afterward MR cisternography and the secondary surgical repair was successfully performed. (Figure 2)

**FIGURE 3.**
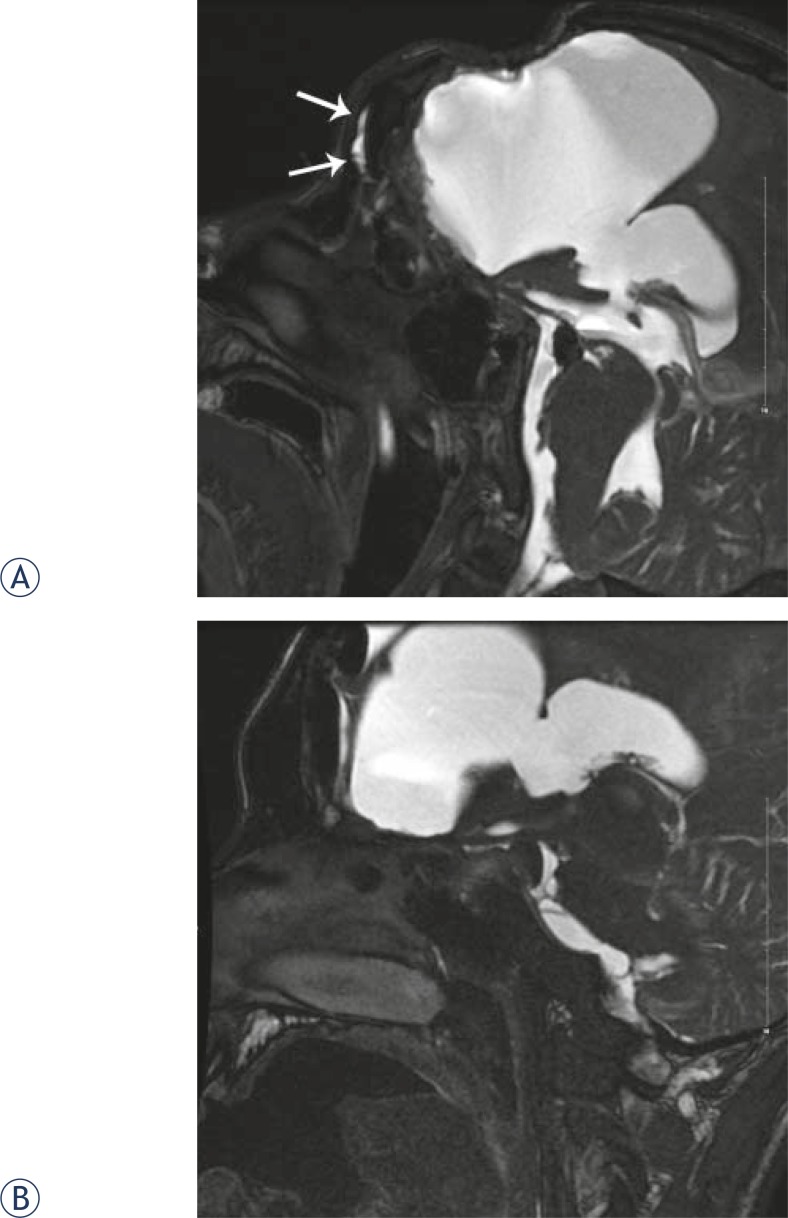
A male patient aged 21 years with severe craniocerebral injury. Surgery was performed to remove part of the frontal bone and contused brain tissue of frontal lobe. One week after surgery, cerebrospinal fluid rhinorrhea was present in left nose. CT scan showed multiple basilar skull fracture and failure in ethmoid sinus repair. MR cisternography showed that high-signal liquid was present in frontal sinus in the prone position (3A) but not in the supine position (3B) in T2-weighted image, thus cerebrospinal leak in frontal sinus was diagnosed. It was observed during surgery that the crack fracture was present in the posterior side of frontal sinus with damaged *dura mater*, which communicated with nasal cavity, and rhinorrhea disappeared after the surgery.

In 11 patients who underwent MR cisternography, 14 sites of cerebrospinal leak were suspected before the surgery. According to the surgical exploration, there were 15 sites of cerebrospinal leak, while 1 site was missed in the MR cisternography. The accuracy was 93% (Table 1). Intermittent cerebrospinal leak was present in the patients missed diagnosed. The diagnostic rate of intermittent and non-active cerebrospinal leak by MR cisternography was not high (Figure 3).

It was demonstrated by surgery that 36 sites of cerebrospinal leak communicated with the paranasal sinus defects, the tympanic cavity, etc, among which 14 sites were in the madreporite, 10 in the frontal sinus and 8 in the sphenoidal sinus. Tympanic cavity of middle ear communicated with defects in petrous and temporal bones in 3 cases and with petrous in 1 case. *Dura mater* was usually thickened and immersed into fracture gap, which was always irregular and slit-like (Table 1).

## Discussion

The severe traumatic brain injury always results in multiple basilar skull fracture, and cerebrospinal leak is one of the most important complications. Cerebrospinal leak may be complicated with bacterial meningitis, and sometimes encephalitis and cerebral abscess.^4^ Although cerebrospinal leak in some cases recovers spontaneously, long-term cerebrospinal leak increases the risk of intracranial infection greatly, with an incidence as high as 40%.^5^ Therefore, the surgical repair is usually required to treat cerebrospinal leak. Location of cerebrospinal leak is essential for the successful surgical repair, especially in patients with cerebrospinal leak after intranasal mini-invasive repair surgery. Many examinations have been applied to locate the cerebrospinal leak, such as radioactive cisternography, which is now seldom used because of the radioactivity, time-consuming, invasion, mild risk, low diagnostic rate and relatively high false positive rate.^2^ With the progress of CT, CT cisternography significantly improves the diagnostic rate of cerebrospinal leak site. Its diagnostic rate is as high as 92% in diagnosing active cerebrospinal leak, while it is just 40% in diagnosing intermittent or non-active cerebrospinal leak.^6^ The application of CT cisternography is also restricted because of lumbar puncture induced infection, hemorrhage and hypotensive cranial pressure headache.

With the rapid popularity of multiple-row high-resolution CT 7, it has currently been regarded as a routine examination and preferred the method for location of cerebrospinal leak because of the safety and convenience. A layer thickness of 0.5 mm is routinely chosen in the modern CT examination and the image reconstruction can be accomplished in random planes^8^,^9^, thus the diagnostic rate of cerebrospinal leak by high-resolution CT is much higher than CT cisternography.^10^ In the present study, 64-row high-resolution CT was applied for thin-layer scan with a layer thickness of 0.625 mm in coronal plane and the multiplanar reconstruction was performed in the scan region to search the leak site. Many basilar skull defects and crack fractures were found out in these 12 patients, but it was difficult to identify which defect was the real cerebrospinal leak site and several wrong or missed diagnoses were made. Several previous studies were performed to evaluate the role of high-resolution CT in identifying cerebrospinal leak site, but the study reporting the leak site location in complicated basilar skull fracture is rare. Many factors made it hard to locate the leak site, including the complication of basilar skull fracture induced by severe traumatic brain injury, thickening *dura mater* in injury site and injury of paranasal sinus. It was easy for CT to find out suspected leak site, but it remained to be difficult to correctly locate the leak site, which depended on the experience and good communication between radiologists and physicians.

Another diagnostic approach was MRI, which is widely used in the case of brain or spinal injuries.^6^,^11^,^12^ Cerebrospinal fluid is present as high signal in T2-weighted spinecho sequence with fat saturation and cerebrospinal leak site is visualized by a non-invasive method. Meanwhile, cephalocele and meningocele are also easily diagnosed. The intrathecal injection of contrast media may also increase the diagnostic rate of MRI cisternography.^11^ Cerebrospinal leak site can also be visualized by changing the position of patients to compare the distribution of cerebrospinal fluid in cranium and paranasal sinuses in different positions.^6^ In the present study, 14 sites of cerebrospinal leak were diagnosed in 11 patients who underwent MRI cisternography and 15 sites were diagnosed by the surgical exploration. The accuracy was as high as 93%. The correct diagnosis was made in 10 out of the 11 patients. Cerebrospinal leak was non-active in the other patient, and the inappropriate timing for MRI cisternography might be the cause of the negative result.

The principle of MR cisternography is similar to CT cisternography and radionuclide cisternography that basilar skull site of cerebrospinal leak was determined by observing current direction of cerebrospinal fluid. Therefore, MR cisternography is good at diagnosing active cerebrospinal leak, while its diagnostic rate was significantly reduced in diagnosing non-active or intermittent cerebrospinal leak.^6^ Trauma induced basilar skull fracture is usually very severe and most of cerebrospinal leaks are active. Therefore, MR cisternography showed high accuracy in our present study. Moreover, MR cisternography has a high demand for positions, and changing position according to the menifestations of patients may improve the diagnostic rate^3^, which requires participation and good communication between physicians and radiologists. MR cisternography after the intrathecal administration of gadopentate dimeglumine represents an effective and minimally invasive method for evaluating suspected cerebrospinal fluid (CSF) fistulas along the skull base. It provides multiplanar capabilities without risk of radiation exposure and is an excellent approach to depict the anatomy of CSF spaces and CSF fistulas.^13^ Brain injury induced by traffic accidents is always accompanied by severe injury in other sites of the body^14^, thus the position of patients may be strictly restricted. One kind of MRI equipment accommodating multiple positions of patients may solve this problem greatly.

High-resolution CT and MRI cisternography were compared systematically to evaluate their roles in diagnosing cerebrospinal leak site. The sensitivity, specificity and accuracy of high-resolution CT were 92%, 100% and 93%, while that of MR cisternography were 87%, 100% and 89% respectively.^6^ It seemed that high-resolution CT was better than MR cisternography and CT showed details of bone defects better. In the present study, the accuracy of MR cisternography was higher than CT. The reasons were listed as follows. First, less multiple basilar skull fracture was included in our study, which made it difficult to diagnose by CT because of the complicated fracture line. Second, most of cerebrospinal leaks in our present study were active, which might increase the diagnostic accuracy of MR. But CT scan has an advantage of visualizing skull defects better in the surgical repair, especially in providing evidence for intranasal endoscopic repair surgery.^5^ Advantages of MR including dynamical display of cerebrospinal fluid flow when changing the position and the correct diagnosis of active leak. But the diagnostic rate of MR in non-active leak is low and MR cannot show basilar skull defect in details. Preoperative CT scan is always required in leak region. Thus, the two examinations have their own advantages and can be applied complementing each other when necessary. We suggested that multiple-row high-resolution CT examinations should be used to locate the suspected leak sites in multiple basilar skull fracture induced by traumatic brain injury. MR cisternography was not necessary if the distributions of leak sites could be involved in one surgical exploration. Cerebrospinal leak sites would be determined by explorations in sequence. When the distributions of leak sites could not be involved in one surgical exploration, MR cisternography should be performed to determine the location which warranted repair in preference.

In conclusion, in multiple basilar skull fracture induced by traumatic brain injury, high-resolution is preferred in identifying multiple basilar skull defects and fractures, but on the other hand, this may lead to difficulty in diagnosing the real cerebrospinal leak sites. MR cisternography shows a current flow of cerebrospinal fluid and thereby determines the leak sites, which covers the insufficiency of high-resolution CT. But it is difficult for MR cisternography to diagnose the sites of non-active cerebrospinal leak, and the diagnosis of non-active leak remains to be further investigated.

## Figures and Tables

**TABLE 1.** Coincidence ratio of CT and MRI examinations with the surgical exploration in diagnosing sites of cerebrospinal leak

**Sites**	**Number**	**CT**			**MR**		
	
		**Coincidence with surgery**	**Missed diagnosis**	**Wrong diagnosis**	**Coincidence with surgery**	**Missed diagnosis**	**Wrong diagnosis**
Ethmoid bone	14	8		2	5	1	
Frontal sinus	10	6	1	1	3		
Sphenoid bone	8	3	1	1	5		
Petrous bone	3	1			1		
Temporal bone	1	1			0		
	
Accuracy			90.48%			93%	
